# High and fast adsorption of Cd(II) and Pb(II) ions from aqueous solutions by a waste biomass based hydrogel

**DOI:** 10.1038/s41598-020-60160-w

**Published:** 2020-02-24

**Authors:** Mingyue Zhang, Quanyu Yin, Xiaoming Ji, Fangling Wang, Xia Gao, Mingqin Zhao

**Affiliations:** College of Tobacco Science, Flavors and Fragrance Engineering & Technology Research Center of Henan Province, Henan Agricultural University, Zhengzhou, 450002 P.R. China

**Keywords:** Pollution remediation, Polymer characterization, Polymer synthesis

## Abstract

A waste biomass based hydrogel soybean residue-poly(acrylic acid) (SR–PAA) was prepared through a fast one-step reaction by UV radiation technology. SR–PAA was used to remove Cd(II) and Pb(II) ions from aqueous solutions. Effect of pH value, temperature, initial concentration, contact time, competitive ions in the solutions on metal ions adsorption and desorption/regeneration capacity of SR–PAA was discussed in detailed. It was found that the adsorption equilibrium was achieved within 20 min, and maximum adsorption for Cd(II) and Pb(II) ions were 1.43 and 2.04 mmol g^−1^, respectively. Besides, adsorption thermodynamic analysis indicates that the process of Cd(II) and Pb(II) ions adsorption was spontaneous, feasible and exothermic in nature. And experimental data fitted the pseudo-second-order and Freundlich isotherm model well. Moreover, XPS spectra analysis proves that the metal ions were adsorbed on SR–PAA due to the interaction of carboxyl, hydroxyl and amine with these ions as ionic bond, coordination bond and electrostatic interaction.

## Introduction

Increasing industrialization has brought great challenges to the environment. Like China, many countries face more and more enormous environmental problems, especially water pollution caused by heavy metal ions^1–3^. Among various heavy metals, Cd(II) and Pb(II) have strong toxicity, which can damage to animals and human bodies seriously though the food chain. The report from United States Environmental Protection Agency showed that cadmium can cause respiratory cancers, for example, lung carcinoma^4^, and lead can cause cognitive dysfunction in children, hypertension, immune system and reproductive system diseases^5^. Consequently, it is particularly necessary to remove the heavy metal ions from the wastewater before discharge it into the environment.

Adsorption is a common method to remove various heavy metal ions from the wastewater. Many kinds of adsorbents, including activated carbon^6^, inorganic minerals^7^, biomass adsorbents^8–10^, and polymer^11–14^, are used to remove the metal ions from the wastewater. Metal ions adsorption capability of an adsorbent is mainly controlled by the surface active sites (functional groups such as carboxyl, hydroxyl, amino and hydrosulphonyl) of the adsorbent^15^. The metal ions that contact with the adsorbent surfaces may be attached to the surfaces of the adsorbent according to physical or chemical interaction. Then those metal ions can be adsorbed on the adsorbent by ion exchange, coordination interaction, electrostatic interaction and physical adsorption, which are considered to be the main mechanism of most adsorbents to remove metal ions. In particular, it has been reported that the group of carboxyl, hydroxyl and amino are extremely advantageous to the metal ions removal from various aqueous solutions^6,9,16–19^. Hence, many researchers are interested in the functional groups modified adsorbents to enhance the capability of metal ions removal. However, an efficient, low-cost, easily obtained and environmental friendly adsorbent is very necessary for the processing of large amounts of sewage. Waste plant biomass as a renewable natural resource, has rich production and low price. Soybean residue, as a main byproduct of soybean processing such as bean curd and soymilk, is a sort of cheap, abundant, green environmental and reproducible natural resource. Soybean residue contains high amounts of cellulose, hemicellulose and protein molecules. The previous literature has proven that hydroxyl and amino groups contained in these molecules can be modified chemically^20–24^, which can further improve the adsorption capacity for metal ions, simultaneously reduce the cost of metal ions removal. Soybean residues are rarely used to synthesize hydrogels to study the removal of metal ions except for the research group of the author. Therefore, we boldly assume that a composite polymer material with high metal ions adsorption can be obtained through carboxylic acid modified soybean residues.

In this work, a novel biomass based hydrogel soybean residue-poly(acrylic acid) (SR–PAA) with three-dimensional network structure was prepared through a simple one-step polymerization reaction via UV radiation. SR–PAA was used as adsorbent to remove Cd(II) and Pb(II) in aqueous solutions. Effect of pH value, temperature and initial concentration, competitive ions, and contact time on Cd(II) and Pb(II) adsorption was discussed. We found that after chemical modification, the hydrogel had higher and faster capacity for adsorption of Cd(II) and Pb(II) ions than soybean residue. In addition, adsorption thermodynamic, isotherms and kinetics were used to fit the experimental data. The adsorption mechanism was discussed according to XPS spectra analysis.

## Results

### Preparation of SR–PAA

**Figure 1:**
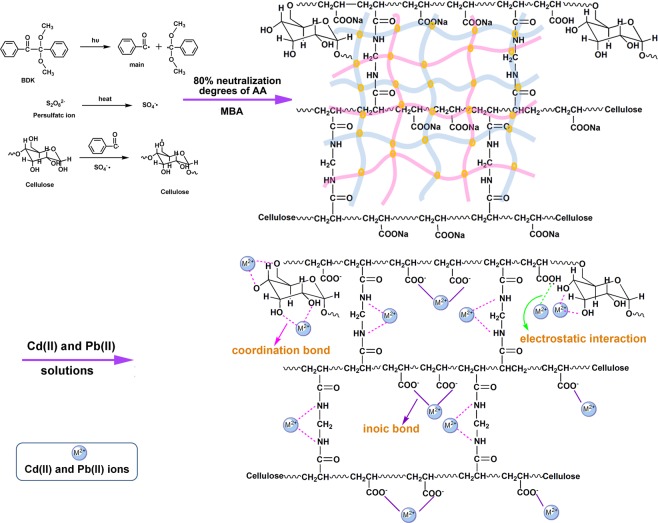
Schematic diagram of SR–PAA synthesis and adsorption of Cd(II) and Pb(II) ions on the hydrogels.

The synthesis of SR–PAA was presented in Fig. 1. Mechanism consisted of three steps: Firstly, under the UV lamp, the initiators decomposed into primary radicals. Then these primary radicals swap out H from –OH (Mainly came from cellulose) in SR to form alkoxy radicals. Secondly, alkoxy radicals reacted with monomers (80% neutralization degrees of AA) to form new radicals. And those radicals reacted with other monomers leading to the chain growth. At the same time, vinyl groups containing in the crosslinker reacted with the chains to form a cross-linked network structure. Finally, polymer chain terminated when the monomers were depleted.

**Figure 2:**
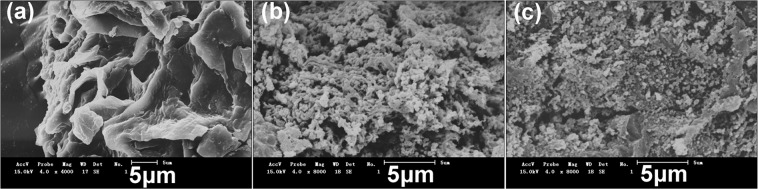
SEM images of (**a**) SR–PAA; (**b**) SR–PAA–Cd and (**c**) SR–PAA–Pb.

The morphology of SR–PAA, SR–PAA–Cd and SR–PAA–Pb were observed by SEM. As shown in Fig. 2, the surface of SR–PAA–Cd and SR–PAA–Pb were entirely different with SR–PAA, which were loose, porous and covered with more and smaller slices.

**Figure 3:**
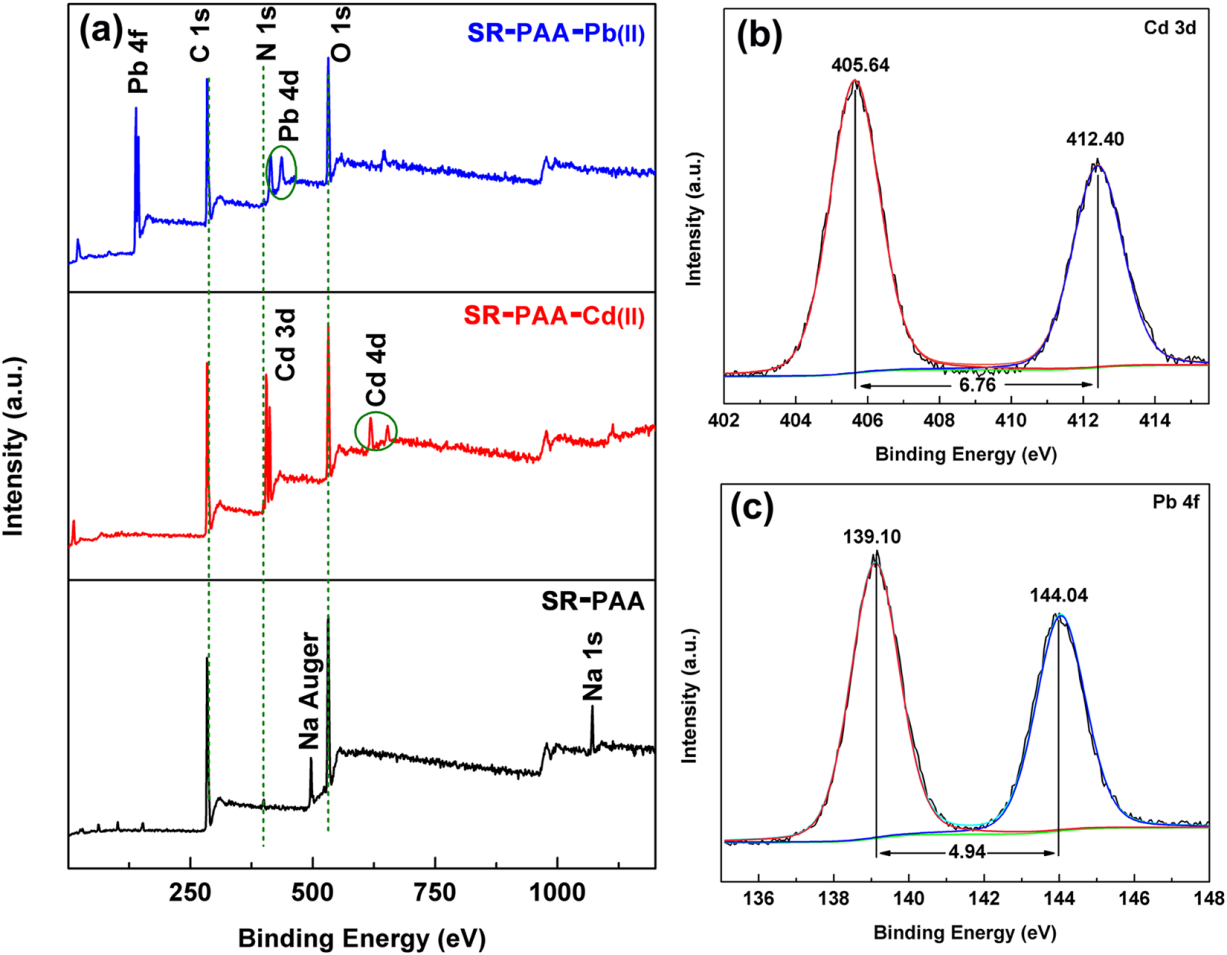
(**a**) XPS spectra of SR–PAA, SR–PAA–Cd and SR–PAA–Pb; XPS spectra of (**b**) Cd 3d of SR–PAA–Cd and (**c**) Pb 4 f of SR–PAA–Pb.

In order to analyze the surface composition of SR–PAA and the interaction with metal ions, the hydrogels before and after adsorption of Cd(II) and Pb(II) were characterized by XPS. As shown in Fig. 3(a), the peaks of C1s, O1s, N1s and Na Auger (497 eV) were observed in SR–PAA, while after adsorption of metal ions, the peak of Na Auger disappeared, and new obvious peaks of cadmium (Cd 3d and Cd 4d) and lead (Pb 4 f and Pb 4d)^25,26^ appeared in the XPS spectra of SR–PAA–Cd and SR–PAA–Pb, respectively, which indicated that the Cd(II) and Pb(II) metal ions were successfully adsorbed on the SR–PAA hydrogel, besides, –(COO)_2_Cd and –(COO)_2_Pb were formed by ion exchange of Cd^2+^and Pb^2+^ ions in solution with –COONa on SR–PAA (Fig. 1).

The Cd 3d and Pb 4 f spectra of SR–PAA–Cd and SR–PAA–Pb hydrogels with curve–fitting were depicted in Fig. 3(b,c), respectively. As shown in Fig. 3(b), the Cd 3d5/2 and Cd 3d3/2 peaks appeared at 405.64 and 412.40 eV, respectively. The difference value of two peaks was 6.76 eV, which were consistent with the standard value^12^. The peak of 405.64 eV was ascribed to Cd^2+^ interaction with the oxygen in C–O and O–C–O, which led to a decrease of the BE of Cd 3d5/2. The peak of 412.40 eV can be ascribed to Cd^2+^ interaction with the oxygen in –COO^−^, forming ionic bond. After adsorption of Pb^2+^ ion, BE of Pb 4f7/2 and Pb 4f5/2 peaks appeared at 139.10 and 144.04 eV, respectively, corresponding to Pb^2+^ interaction with the oxygen in C–O, O–C–O and –COO^−^, respectively (Fig. 1)^12^.

**Figure 4:**
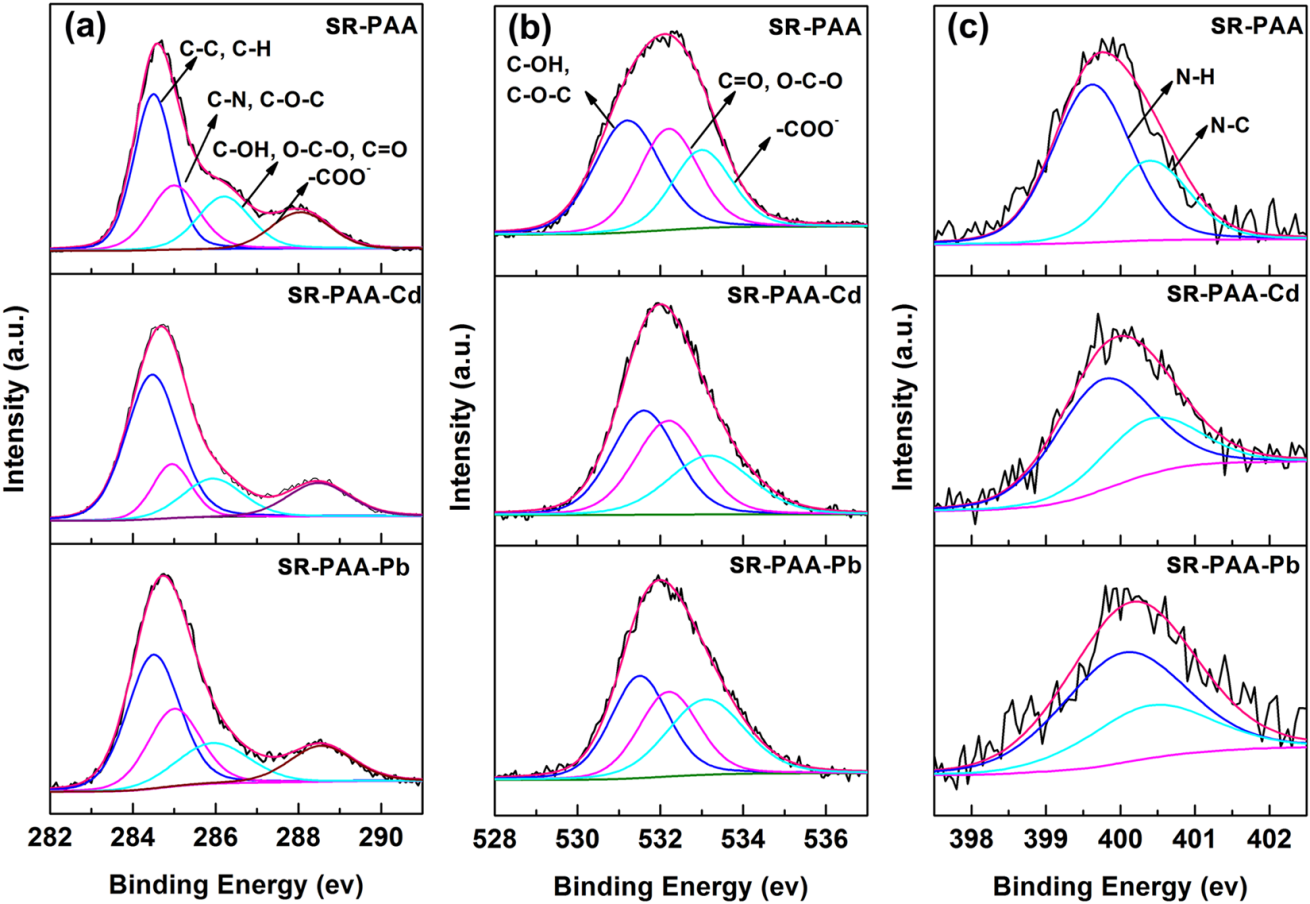
XPS spectra of (**a**) C 1s, (**b**) O 1s and (**c**) N 1s peaks for SR–PAA, SR–PAA–Cd and SR–PAA–Pb hydrogels.

The C 1s, O 1s and N 1s spectra had been deconvoluted into four, three and two peaks by using Lorentzian-Gaussian fit, respectively (Fig. 4 and Supplementary file Table S1). For C 1s of SR–PAA, the binding energy (BE) of 284.50 eV belonged to C–C and C–H, with atomic fractions (AF) of 27.78%, 285.00 eV belonged to C–N, and C–O–C, with AF of 15.23%, 286.20 eV belonged to C–OH, C=O and O–C–O, with AF of 12.44% and 288.05 eV belonged to –COO^−^, with AF of 9.54%. After adsorption of Cd(II) and Pb(II), the first two peaks of C 1s remained almost no change, but the peak at 286.20 eV all shifted to 285.93 eV, which was due to the interaction of Cd(II) and Pb(II) ions with C–OH. The formation of metal complexes C–O–Cd^+^ and C–O–Pb^+^ made the electrons transfer to carbon atom via oxygen atom, which led to their corresponding BE decreased. The ion exchange action of metal ions with –COONa and –COOH on polymer chains made the peak at 288.05 eV shifted towards higher energy.

Compared with O 1s and N 1s of SR–PAA, there were similar minor changes after adsorption of metal ions. From the O 1s spectrum, peak 1: 531.20 eV, corresponding to C–OH and C–O–C with no metal ions interaction. After adsorption of Cd(II) and Pb(II), the peak shifted to 531.59 and 531.50 eV, respectively, corresponding to C–O with metal ions interaction. Peak 2: 532.20 eV, corresponding to C=O and O–C–O. Peak 3: 533.00 eV, corresponding to –COO^−^^27^, after adsorption of metal ions, the peak shifted to 533.23 and 533.10 eV, respectively, corresponding to –(COO)_2_Cd and –(COO)_2_Pb, respectively. For the O 1s spectrum, 399.62 eV was corresponding to N–H, after adsorption of metal ions, it shifted to 399.76 and 400.05 eV, respectively. It was due to the interaction of nitrogen atom with metal ions^27^. Ion–exchange interaction, electrostatic interaction and coordination occurred between Cd(II), Pb(II) ions and the polymer chains (Fig. 1), which led to the changes of carbon, oxygen and nitrogen electronic environment.

### Effect on Cd(II) and Pb(II) equilibrium adsorption

#### Effect of pH value

**Figure 5:**
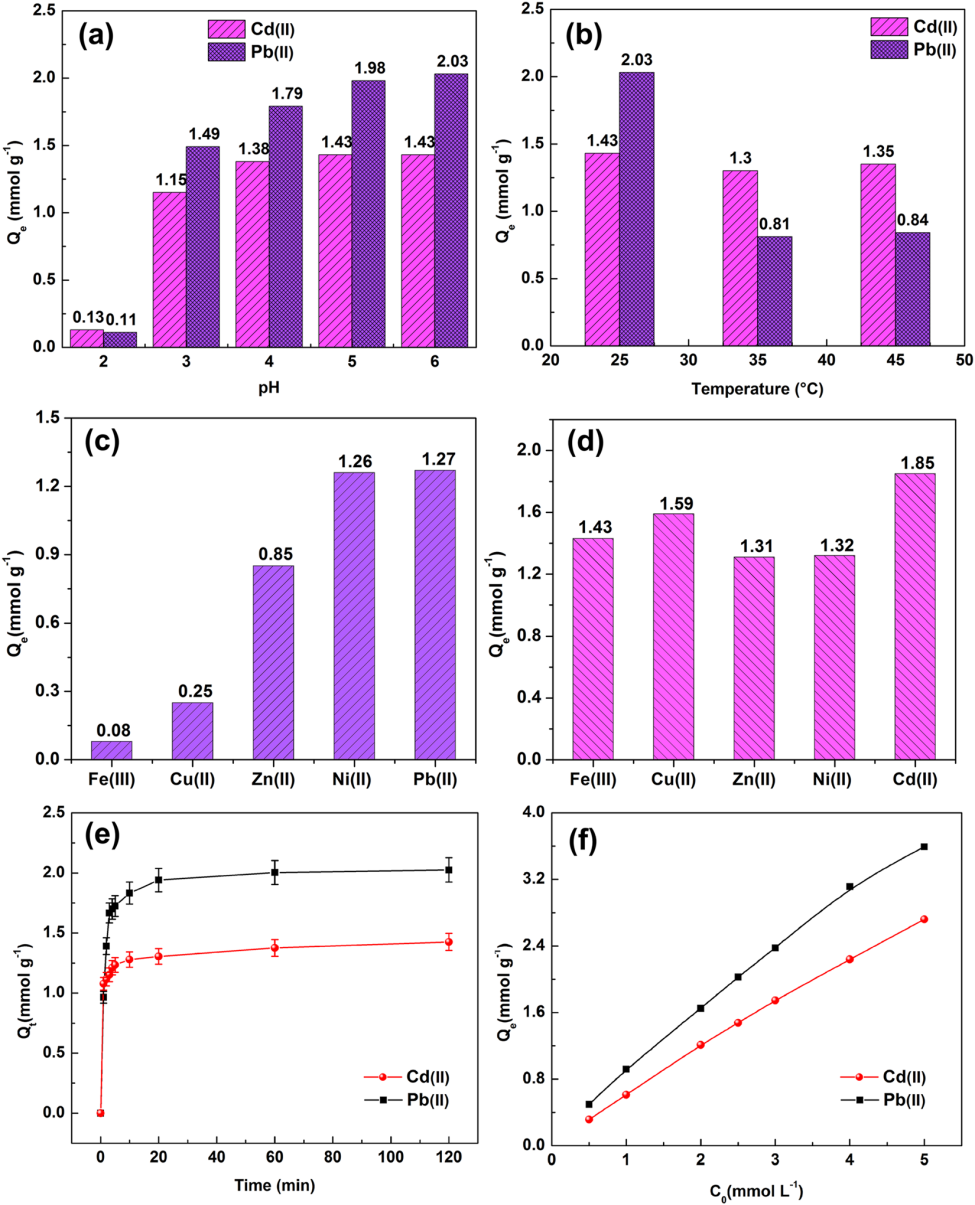
Effect of (**a**) pH value and (**b**) temperature on Cd(II) and Pb(II) ions adsorption; effect of competitive ions on (**c**) Cd(II) and (**d**) Pb(II) adsorption; (**e**) adsorption time and (**f**) initial concentration on Cd(II) and Pb(II) ions adsorption.

The pH value of the solutions direct influences the removal efficiency of metal ions. As shown in Fig. 5(a) with pH increasing, the equilibrium adsorption of metal ions on SR–PAA enhanced. For the solution with pH 2, 3, 4, 5 and 6 (25 °C), the equilibrium adsorption of Cd(II) was 0.13, 1.15, 1.38, 1.43 and 1.43 mmol g^−1^, respectively, and the equilibrium adsorption of Pb(II) was 0.11, 1.49, 1.79, 1.98, 2.03 mmol g^−1^, respectively. At above mentioned pH values (except at pH 2), the adsorption of SR–PAA to Pb(II) was more than Cd(II). The adsorption of SR–PAA to Cd(II) and Pb(II) increased with the increase of pH value. It was because when pH was low, more hydrogen ions were contained in the system, and took up more active adsorption sites, and the adsorption of metal ions was reduced accordingly^13,28^. In other words, carboxylate groups of p(acrylic acid) converted to carboxylic acid groups due to H binding at acidic pHs, ie below pK_a_, resulting in the reduction of metal ions adsorption. When pH value increases, for example at 6, the decrease of hydrogen ions released more adsorption sites, which was conducive to the adsorption of metal ions.

#### Effect of temperature

The effect of solutions temperature on Cd(II) and Pb(II) ions adsorption was examined. As shown in Fig. 4(b), at 25 °C, 35 °C and 45 °C, the equilibrium adsorption of Cd(II) were 1.43, 1.41 and 1.39 mmol g^−1^, respectively, and that of Pb(II) were 2.03, 1.93 and 1.88 mmol g^−1^, respectively. The adsorption declined as the temperature rising (25 °C–45 °C), which may be due to that the adsorption of metal ions on SR–PAA was exothermic process, low temperature was advantageous to the metal ions adsorption.

#### Effect of competitive ions

The effect of other competitive ions in aqueous solutions on Cd(II) and Pb(II) adsorption was investigated. As can be seen from Fig. 5(c,d), after respectively adding the same concentration (2.5 mmol L^−1^) of competitive ions Fe(III), Cu(II), Zn(II), Ni(II) and Pb(II), the adsorption of Cd(II) ion on SR–PAA decreased to 0.08, 0.25, 0.85, 1.26 and 1.27 mmol L^−1^, respectively, while after adding Fe(III), Cu(II), Zn(II), Ni(II) and Cd(II) ions, the adsorption of Pb(II) ion on SR–PAA dropped to 1.43, 1.59, 1.31, 1.32 and 1.85 mmol L^−1^, respectively. The influence of the competitive ions on Cd(II) adsorption followed the sequence: Fe(III) > Cu(II) > Zn(II) > Ni(II) > Pb(II). This order agreed well with the first stability constant of the associated metal acetate and metal hydroxide. From “Lange’s handbook of chemistry”, it can be found that the first stability constant of the associated metal acetate of Fe(III), Cu(II), Zn(II), Ni(II) and Pb(II) were 3.2, 2.16, 1.50, 1.12 and 2.52, respectively [M^n+^ + -COO^−^ ↔ M(COO)^n−^], and there first stability constant of associated metal hydroxide were 11.87, 7.0, 4.4, 4.97 and 7.82, respectively [M^n+^ + OH^−^ ↔ M(OH)^n−^]. Besides, metal electronegativity and effective ionic radius may also had effect on the competitive adsorption. [Metal electronegativity was 1.83, 1.90, 1.65, 1.91, 1.8 and 1.69; effective ionic radius was 55, 57, 60,55 98 and 148 pm for elements of Fe(III), Cu(II), Zn(II), Ni(II) and Pb(II), Cd(II), respectively]. The influence of the competitive ions on Pb(II) adsorption followed the order: Zn(II) > Ni(II) > Fe(III) > Cu(II) > Cd(II).

#### Effect of adsorption time

Fig. 5(e) showed the effect of contact time on metal adsorption. The adsorption process can be divided into three stages: the first stage, 0–3 min, rapid adsorption process, the adsorption of Cd(II) and Pb(II) were 1.15 and 1.67 mmol g^−1^, which reached 80.42 and 82.27% of the equilibrium adsorption, respectively. The second stage, 4–10 min, slow adsorption, the adsorption of Cd(II) and Pb(II) were 1.28 and 1.83 mmol g^−1^, reaching 89.51 and 90.15% of the equilibrium adsorption, respectively, which illustrates SR–PAA had a fast adsorption rate of Cd(II) and Pb(II) ions. The third stage, 10–20 min, nearly equilibrium process, the contribution was approximately 9% of the equilibrium adsorption. The equilibrium adsorption of Cd(II) and Pb(II) ions on SR–PAA was 1.43 and 2.03 mmol L^−1^, respectively, which was 6.15 and 47.67 times of that on SR (Table 1). This suggests that chemical modification can greatly improve adsorption capacity of metal ions, having the benefit for metal ions removal from aqueous solutions. In addition, adsorption capacity (mmol g^−1^) for Cd(II) and Pb(II) ions on SR-PAA was compared with various other adsorbents in Table 1. It is quite surprising that SR-PAA possess higher metal ions adsorption, and reach adsorption equilibrium quicker than most other adsorbents. For its high and fast metal ions adsorption capacity, low-cost and easily obtained properties, it could be considered a promising adsorbent for the metal ions removal from large amounts of sewage.

**Table 1:** Comparison of Pb(II) and Cd(II) ions adsorption capacity (mmol g^−1^) on SR-PAA and equilibrium adsorption time with various other adsorbents.

Adsorbents	Q_e_ (mmol g^−1^)		Contact time (min)^a^	T (°C)	pH	Reference
	Pb(II)	Cd(II)				
Raw maize stover	0.09	—	60	25	5	^39^
HNO_3_ treated maize stover	0.13	—	60	25	5	^39^
NaOH modified agaricus bisporus	0.42	—	—	25	*5.5*	^40^
Orange peel biochar (OP-BC)	0.13	—	15	25	*5–6*	^41^
Amino siloxane oligomer-linked graphene oxide	1.51	—	300	30	4.0–5.0	^42^
Natural Artemia CS	1.54	—	2	25	6.4 ± 0.1	^43^
Snowflake-shaped zno@sio_2_@Fe_3_O_4_/C	0.46	—	180	—	7	^44^
MWCNT-PDA hybrid aerogels^b^	1.69	—	600	25	6	^45^
Biochars derived from Switchgrass (SW450)	0.05	—	1440	22		^46^
Fe_3_O_4_@PMAA yolk–shell microspheres	2.48	—	240	25	6	^47^
Biochars originated from Grape stalks	2.87	—	1440	22 ± 1	5	^48^
Biochars originated from wheat straws	1.32	—	1440	22 ± 1	5	^48^
Biochars originated from grape hulls	0.86	—	1440	22 ± 1	5	^48^
Cotton derived porous carbon (CDPC)	0.10	0.07	overnight	25	5 (Pb), 6(Cd)	^29^
Cotton derived porous carbon oxide (CDPCO)	0.54	0.36	overnight	25	5 (Pb), 6(Cd)	^29^
Chestnut bur	0.20	0.15	360	20–25	4	^49^
Functionalized yeast cells	0.56	0.38	—	25	5	^50^
Mercapto-modified coal gangue (CG-SH)	0.94	0.76	>250(Pb), >200(Cd)	25	5.5	^51^
Fe(III)—pomegranate peel carbon		0.22	120	—	6.36	^52^
Sunflower head carbon (SHC)		0.25	180	—	6	^53^
Sunflower stem carbon (SSC)		0.32	180	—	6	^53^
Posidonia oceanica biomass		0.23	30	—	6	^54^
Magnesium chloride-modified Lentinula edodes		0.46	120	—	5	^55^
Cu_3_(BTC)_2_–SO_3_H		0.79	10	—	6	^56^
SR	0.33	0.03	60	—	6	This work
SR-PAA	2.03	1.43	20	—	6	This work

^a^Equilibrium adsorption time.

^b^Graphene/polydopamine modified multiwalled carbon nanotube hybrid aerogels.

#### Effect of initial concentration

Effect of initial concentration of Cd(II) and Pb(II) ions on metal adsorption was investigated (Fig. 5(f)). The results showed that the equilibrium adsorption of Cd(II) and Pb(II) ions increased with the increase of initial concentration. When the initial concentration was 0.50, 1.00, 2.00, 3.00, 4.00 and 5.00 mmol L^−1^, adsorption of Cd(II) was 0.32, 0.61, 1.21, 1.48, 1.75, 2.23 and 2.72 mmol g^−1^, respectively, and that of Pb(II) was 0.49, 0.92, 1.65, 2.03, 2.38, 3.11 and 3.59 mmol g^−1^, respectively. SR–PAA exhibited better removal efficiency of Pb(II) than Cd(II).

### Adsorption thermodynamic

The thermodynamic parameters were calculated by Eqs. 2–5, and shown in Table 2. As shown in Table 2, the values of ΔG were all negative, and increased when the temperature raised from 298 K to 318 K, which indicated that the Cd(II) and Pb(II) ions adsorption process on SR–PAA was spontaneous and feasible, and high temperature was not conducive to the adsorption^29^. Moreover, the absolute values of ΔG at 298, 308 and 318 K for adsorption of Pb(II) were all greater than that for Cd(II), which showed that it was more conducive for Pb(II) adsorption. It also provided a theoretical basis for the experiment that the adsorption of Pb(II) (1.85 mmol g^−1^) was higher than that of Cd(II) (0.85 mmol g^−1^) on SR–PAA hydrogel (can be seen in Fig. 4c). The negative value of ΔH illustrates that the adsorption process of Cd(II) and Pb(II) ions on SR–PAA was exothermic in nature^30,31^. That was also the reason equilibrium adsorption of Cd(II) and Pb(II) decreased as the rising of solution temperature (Effect of temperature). The value of ΔS were −20.31 and −6.38 J mol^−1^ K^−1^ for adsorption of Cd(II) and Pb(II) ions, which reflected that the randomness at the interface of SR–PAA hydrogel and solution was reduced during the adsorption process.

**Table 2:** Thermodynamic parameters of Cd(II) and Pb(II) ions adsorption on SR–PAA.

Ions	T (K)	ΔG (kJ mol^−1^)	ΔH (kJ mol^−1^)	ΔS (J mol^−1^K^−1^)	R^2^
Pb(II)	298	−2.16	−8.11	−20.31	0.9376
	308	−1.96			
	318	−1.75			
Cd(II)	298	−0.17	−2.04	−6.38	0.9992
	308	−0.11			
	318	−0.04			

### Adsorption kinetics

The results of the pseudo-first-order and the pseudo-second-order model were displayed in Table 3, respectively. The correlation coefficients (R^2^) of the pseudo-second-order for adsorption of Cd(II) and Pb(II) were all more than 0.99, which were closer to 1 than that of the pseudo-first-order. Moreover, the equilibrium adsorption of Cd(II) and Pb(II) ions that calculated from the pseudo-second-order (*Q*_*e, cal*_) were 1.43 and 2.04 mmol g^–1^, respectively, which was consistent with the experimental value (1.43 mmol g^−1^ and 2.03 mmol g^−1^). While *Q*_*e, cal*_ from the pseudo-first-order was large difference with experimental data. Based on the above two reasons the adsorption process fitted with the pseudo-second-order model well. Furthermore, it can be seen from the calculated *K*_2_ that the adsorption rate of Cd(II) and Pb(II) ions were 0.7371 and 0.5170 g mmol^−1^ min^−1^, respectively, which revealed that the SR-PAA could remove the metal ions from the liquor fast, and the adsorption rate followed the order Cd(II) > Pb(II).

**Table 3:** Parameters of adsorption kinetics, diffusion kinetic and adsorption isotherms for the adsorption of Cd(II) and Pb(II) on SR–PAA hydrogel.

model	Parameters	Cd(II)	Pb(II)
pseudo-first order	R^2^	0.5310	0.7500
	*K*_1_ (min^–1^)	0.0365	0.0626
	*Q*_*e, cal*_ (mmol g^–1^)	0.96	0.61
pseudo-second order	R^2^	0.9997	0.9999
	*K*_2_ (g mmol^−1^ min^−1^)	0.7371	0.5170
	*Q*_*e, cal*_ (mmol g^–1^)	1.43	2.04
Diffusion mechanism	R^2^	0.9714	0.9963
	k_1_	0.7546	0.4788
	n_1_	0.0596	0.5011
	R^2^	0.9729	0.9955
	k_2_	0.7855	0.7462
	n_2_	0.0581	0.0835
	R^2^	0.9993	0.9509
	k_3_	0.7898	0.8919
	n_3_	0.0492	0.0244
Intra-particle diffusion	R^2^	0.9939	0.9955
	K _i 1_ (mmol g^−1^ min^−1/2^)	0.1010	0.9631
	C_1_ (mmol g^−1^)	0.9757	0.0103
	R^2^	0.9542	0.9997
	K _i 2_ (mmol g^−1^ min^−1/2^)	0.0561	0.1151
	C_2_ (mmol g^−1^)	1.1033	1.4692
	R^2^	0.9826	0.8502
	K _i 3_ (mmol g^−1^ min^−1/2^)	0.0186	0.0131
	C_3_ (mmol g^−1^)	1.2254	1.8891
Langmuir	R^2^	0.9858	0.8984
	*Q*_*max*_ (mmol g^−1^)	10.31	5.78
	K_L_ (L mmol ^−1^)	0.1267	0.7011
Freundlich	R^2^	0.9999	0.9989
	1/n	0.8914	0.6664
	K_F_ (mmol g^−1^)	1.1240	2.2261

### Diffusion kinetic

The fitting curves of adsorption of Cd(II) and Pb(II) ions revealed multi-linearity  (Supplementary file Figure S3), which represented three stages, respectively. As shown in Table 3, the correlation coefficients (R^2^) of all stages for Cd(II) and Pb(II) adsorption were more than 0.95. For adsorption of Cd(II), n_1_, n_2_ and n_3_ were all less than 0.45, conforming to Fickian diffusion, which indicated that the transfer of metal ions to the polymer network was dominant in the process of Cd(II) adsorption. While for adsorption of Pb(II), first stage, n_1_ > 0.45, the process conformed to non-Fickian diffusion, the synergy of medium solution transfer and polymer chain relaxation of the hydrogel network was the cause of Pb(II) adsorption. The last two stages, n_2_ and n_3_ were less than 0.45, conformed to Fickian diffusion.

The results of intra-particle diffusion model (Table 3) showed that K_i 1_ > K_i 2_ > K_i 3_, which indicated that the order of ions diffusion speed was first stage > second stage > third stage. Higher concentration and more adsorption sites made the initial phase having larger diffusion rate, and with the extension of the adsorption time, concentration of the solution down to a lower value and most of the adsorption sites were occupied by the metal ions, which led to an extremely low diffusion rate^32^.

### Adsorption isotherms

The fitted curves of the Freundlich and Langmuir model were provided in Supplementary Information and their parameters were displayed in Table 2.

Compare the R^2^ of Langmuir (0.9858 and 0.8984) and Freundlich model (0.9999 and 0.9989), Freundlich isotherm fitted the experimental data better. In addition, it can be seen from the parameters of Langmuir model, the maximum adsorption capacity were 10.31 and 5.78 mmol g^−1^, K_L_ were 0.1267 and 0.7011 L mmol^−1^ for adsorption of Cd(II) and Pb(II), respectively, which was quite different from the experimental results. K_F_ values of the Freundlich (1.1240 and 2.2261 L mmol^−1^ for adsorption of Cd(II) and Pb(II) agreed well with the experimental results. 1/n form Freundlich isotherm were less than 1, suggesting the SR-PAA hydrogel was favorable to adsorb of metal Cd(II) and Pb(II). Therefore, the analysis results showed that the Freundlich isotherm model was fitted the equilibrium Cd(II) and Pb(II) adsorption better than the Langmuir model.

### Desorption and regeneration of SR-PAA

**Figure 6:**
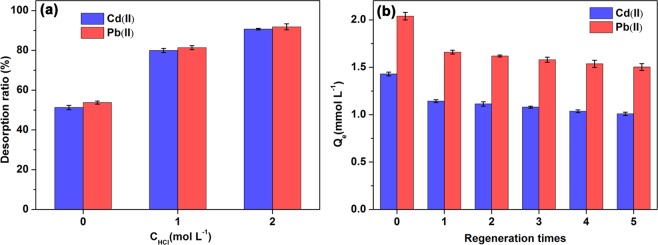
(**a**) The desorption of metal ions by different concentrations of HCl; (**b**) Reusability of SR-PAA during five cycles (The desorption solution was 1 mol L^−1^ HCl).

The desorption and regeneration results of SR-PAA were shown in Fig. 6. As shown in Fig. 6(a), the metal-loaded SR-PAA can be regenerated by pickling. The desorption ratio of metal ions increased with the concentration of HCl. The maximum desorption ratio reached the maximum in 2 mol L^−1^ HCl, which was 90.7% and 91.8% for Cd(II) and Pb(II) ions, respectively. As shown in Fig. 5(b), the adsorption of metal ions decreased the most during the first recycle, which was because that some of the metal ions have not been desorbed by HCl (It can be found from Fig. 6(a) that 20.0% and 18.6% of Cd(II) and Pb(II) ions were undesorbed in 1 mol L^−1^ HCl, respectively). These undesorbed metal ions occupied part of the adsorption sites, resulting in the decrease of metal ion adsorption. The adsorption of metal ions was not much affected during the subsequent adsorption-desorption cycles. After 5 cycles reused, the adsorption for Cd(II) and Pb(II) ions was 1.01 and 1.50 mmol g^−1^, which was 70.6%% and 73.9% of adsorption for the first time, respectively.

## Conclusions

In this study, it was confirmed that soybean residues can be used to synthesize hydrogel SR–PAA. The hydrogel have an effective adsorption of Cd(II) and Pb(II) ions from aqueous solutions, was prepared through a one-step reaction. The main conclusions were as follows:

1. The equilibrium adsorption of Cd(II) and Pb(II) ions were 1.43 and 2.04 mmol g^−1^, respectively.
2. The adsorption capacity of Cd(II) and Pb(II) ions on SR–PAA increased as pH value increased while decreased as temperature increased. The influence of the competitive ions in aqueous solutions on Cd(II) adsorption followed the sequence: Fe(III) > Cu(II) > Pb(II) > Zn(II) > Ni(II), and that on Pb(II) followed the order: Zn(II) > Ni(II) > Fe(III) > Cu(II) > Cd(II).
3. The experimental data fitted the pseudo-second-order and Freundlich isotherm model well.
4. Adsorption thermodynamic analysis indicated that the process of Cd(II) and Pb(II) ions adsorption was spontaneous, feasible and exothermic in nature.
5. XPS spectra showed that the effective Cd(II) and Pb(II) ions removal ability of SR–PAA came from the interaction of carboxyl, hydroxyl and amine in SR–PAA with metal ions in aqueous solutions.
6. SR–PAA exerted good reusability for Cd(II) and Pb(II) ions.

## Materials and Methods

### Materials

Soybean residue were obtained from Zhengzhou, Henan, China, and sieved through 160 mesh steel screen. Acrylic acid (AA) and ammonium persulfate (APS) were purchased from Fuchen Chemical Reagents (Tianjin, China). N, N’-methylenebis(acrylamide) (MBA), benzoin dimethyl ether (BDK), methyl alcohol, sodium hydroxide (NaOH), cadmium nitrate tetrahydrate (Cd(NO_3_)_2_ 4H_2_O), lead nitrate (Pb(NO_3_)_2_), zinc sulfate hexahydrate (ZnSO_4_·6H_2_O), ferric chloride hexahydrate (FeCl_3_·6H_2_O), copper sulfate pentahydrate (CuSO_4_·5H_2_O) and nickel nitrate hexahydrate (Ni(NO_3_)_2_·6H_2_O) were supplied by Sinophorm chemical Reagent Co., Ltd. All the chemical reagents were analytical grade.

### Preparation of SR–PAA hydrogel

2.0 mL 80% neutralization degrees of AA solution (It was obtained through the neutralization reaction of AA and 20 wt % NaOH aqueous solution), certain amount of SR, APS and BDK (0.05 g mL^–1^) and MBA (0.02 g mL^–1^) with mass ratio of AA/SR/MBA/APS/BDK = 100: 60: 0.2: 0.10: 1.25 were mixed thoroughly. After that, the mixtures was transfered into a homemade quartz device (It was airtight and quadrate with quartz glass thickness of 2 mm) with a syringe, and put the device under a 250 W UV lamp (λ = 365 nm) for 10 min, keeping the distance between the mixture and the UV lamp was 15 cm. After reaction, the obtained sample was soaked in 50 mL methyl alcohol for 12 h to remove the unreacted monomer and soluble oligomer. Finally, the hydrogel was dried completely in an oven at 70 °C, crushed and sieved through 80 mesh steel screen.

### Metal ions adsorption measurement

Stock solutions of Cd(II) and Pb(II) solutions (5 mmol L^−1^) were prepared by dissolving Cd(NO_3_)_2_ 4H_2_O and Pb(NO_3_)_2_ in distilled water, respectively. Other concentration solutions were obtained by diluting the stock solution with distilled water. The pH value of solutions was adjusted using 0.1 mol L^−1^ HNO_3_ or NaOH solution. Fresh prepared solutions were utilized for all experiments.

Firstly, 35 mL certain concentration of salt solution and 0.03 g adsorbent was taken into a 50 mL centrifuge tube. Then put the tube in a SHZ-82A thermostatic water bath shaker for certain time (t min) (200 rpm). Secondly, the tube was taken out and centrifuged at 8000 rpm for 5 minutes. Then the supernatant was sucked by a syringe. Finally, the concentration of the solutions before and after the adsorption was determined. Metal ions adsorption capacity of SR–PAA adsorbent was calculated according to Eq. 1^33^.

1 $${Q}_{t}=\frac{({C}_{0}-{C}_{t})V}{m}$$

where C_0_ (mmol L^−1^) was initial concentration of metal ions and C_t_ (mmol L^−1^) was concentration of metal ions after adsorption for *t* minutes. *V* (L) was the volume of the metal ion solution, m (g) was mass of SR-PAA adsorbent.

### Effect of adsorption conditions

Effect of pH value on metal adsorption on SR-PAA was determined by soaking SR-PAA in solutions (2.5 mmol L^−1^) at pH value of 2, 3, 4, 5 and 6 at 25 °C, respectively. Experiments of temperature effect were carried out at 25, 35 and 45 °C, respectively (pH = 6, C_0_ = 2.5 mmol L^−1^). Experiments of initial concentration effect were implemented with C_0_ of 0.50, 1.00, 2.00, 3.00, 4.00 and 5.00 mmol L^−1^, respectively (25 °C, pH = 6). Effect of contact time on metal adsorption experiment was evaluated with soaking time of 1, 2, 3, 4, 5, 10, 20, 60 and 120 min, respectively (25 °C, pH = 6, C_0_ = 2.5 mmol L^−1^). And the hydrogels that adsorption for 120 minutes were recorded as SR–PAA–Cd and SR–PAA–Pb, respectively. Adsorption time of all the experiments was 120 min except that effect of time experiments.

### Adsorption thermodynamic

The adsorption process was analyzed by thermodynamic theory. Thermodynamic parameters were calculated by Eqs. 2–5 ^31,34^. The Gibbs free energy change of the adsorption (ΔG) could be calculated by Eq. 2.

2 $$\Delta G=-\,RT\,\mathrm{ln}\,{K}_{D}$$

where R was the gas constant, and R = 8.314 J mol^−1^K^−1^, T (K) was the absolute temperature. K_D_ was the equilibrium constant, which was calculated by Eq. 3.

3 $${K}_{D}=\frac{{Q}_{e}}{{C}_{e}}$$

where Q_e_ (mmol g^−1^) and C_e_ (mmol L^−1^) were equilibrium adsorption of metal ions and metal ions concentration of solution in equilibrium, respectively. Equation 4 showed the relation of ΔG with the enthalpy change ΔH (kJ mol^−1^) and entropy change ΔS (J mol^−1^ K^−1^) of adsorption process. ΔH and ΔS were calculated from the slope and intercept of lnK_D_ versus 1/T plot (Eq. 5).

4 $$\Delta G=\Delta H-T\Delta S$$

5 $$\mathrm{ln}\,{K}_{D}=\frac{\Delta S}{R}-\frac{\Delta H}{RT}$$

### Adsorption kinetic

The pseudo-first-order and the pseudo-second-order model were established to evaluate the control mechanisms of adsorption kinetics of Cd(II) and Pb(II) ions on SR–PAA. The linear form of pseudo-first-order could be expressed as Eq. 6^30^.

6 $$\mathrm{ln}({Q}_{e}-{Q}_{t})=\,\mathrm{ln}\,{Q}_{e}-{K}_{1}t$$

The linear form of pseudo-second-order kinetic model could be obtained, and showed as Eq. 7^35^.

7 $$\frac{t}{{Q}_{t}}=\frac{1}{{K}_{2}{{Q}_{e}}^{2}}+\frac{t}{{Q}_{e}}$$

where *Q*_*e*_ (mmol g^−1^) was equilibrium adsorption of Cd(II) and Pb(II) ions, *Q*_*t*_ (mmol g^−1^) was the two ions adsorption at adsorption time *t* (min), *K*_1_ (min^−1^) was rate constant of the pseudo-first-order and *K*_2_ (g mmol^−1^ min^−1^) was rate constant of the pseudo-second-order.

### Diffusion kinetic

The Fickian diffusion kinetic and intra-particle diffusion model were used to examine the diffusion mechanism of metal ions into the network of SR–PAA hydrogel.

The logarithmic form of Fickian diffusion kinetic model was shown as Eq. 8^36^.

8 $$\log ({M}_{t}/{M}_{e})=\,\log (k)+n\,\log (t)$$

where *M*_*t*_ and *M*_*e*_ were the mass (mg) of ions adsorbed at time *t* (min) and at equilibrium adsorption, respectively. *k* was a characteristic constant. *n* was the diffusional exponent, describing the diffusion mechanism of metal ions. The parameters of *n* and *k* were calculated by the slope and intercept of linear regression curves of log(*M*_*t*_*/M*_*e*_) versus log(*t*).

The intra-particle diffusion kinetic was presented as Eq. 9^37^.

9 $${Q}_{t}={K}_{i}\cdot {t}^{1/2}+C$$

where K_i_ was the intra-particle diffusion rate constant (mmol g^−1^min^−1/2^) and C was a constant (mmol g^−1^), which was considered be proportional to the thickness of the boundary layer.

### Adsorption isotherms

Freundlich and Langmuir model were used commonly to describe the adsorption isotherms. The Langmuir model and its linearized form were given as Eqs. 10 and 11, respectively^37^.

10 $${Q}_{e}=\frac{{K}_{L}{Q}_{\max }{C}_{e}}{1+{K}_{L}{C}_{e}}$$

11 $$\frac{{C}_{e}}{{Q}_{e}}=\frac{1}{{Q}_{\max }{K}_{L}}+\frac{{C}_{e}}{{Q}_{\max }}$$

where Q_max_ (mmol g^−1^) was the maximum adsorption capacity, C_e_ (mmol L^−1^) was the concentration of Cd(II) and Pb(II) ions in solutions at equilibrium, Q_e_ (mmol g^−1^) was the equilibrium adsorption of Cd(II) and Pb(II) ions and K_L_ (g mmol^−1^) was the Langmuir adsorption constant.

The Freundlich model and its logarithmic form were given as Eqs. 12 and 13, respectively^38^.

12 $${Q}_{e}={K}_{F}{{C}_{e}}^{\frac{1}{n}}$$

13 $$\mathrm{ln}\,{Q}_{e}=\frac{1}{n}\,\mathrm{ln}\,{C}_{e}+\,\mathrm{ln}\,{K}_{F}$$

where K_F_ (mmol g^−1^) was an indicative constant for adsorption capacity of the adsorbent and 1/n (0 ~ 1) could respond the adsorption intensity or surface heterogeneity of the adsorbent.

### Desorption and regeneration of SR-PAA

Desorption studies were carried out by using 50 mL of HCl (0.1, 1 and 2 mol L^−1^) as desorption solution. After the equilibrium study, metal-loaded adsorbent was obtained and washed with deionized water, and then dried to the constant weight. Then 0.1 g adsorbents were immersed in 50 mL HCl. The mixed liquor was stirred at room temperature for 2 h and then filtered through a centrifuge. The metal concentrations in the filtrate were determined to analysis metal ions desorbed. The metal-desorbed SR–PAA would subsequently be used for the next cycle of adsorption/desorption experiment to evaluate its regeneration ability. The desorption ratio was calculated from Eq. 14.

14 $$Desorption\,ratio( \% )=\frac{{m}_{de}}{{m}_{ad}}\times 100 \% $$

where m_de_ (mg) and m_ad_ (mg) were the amount of desorbed metal ions and adsorbed ions in the previous cycle, respectively.

### Characterization

SEM were recorded on a SSX–550 SEM instrument (SHIMDZU) after coating the samples with gold using a ETD–2000 auto sputter coater (ETDC, Ltd.). The concentration of Cd(II) and Pb(II) in aqueous solution was quantified by using Inductive Coupled Plasma Emission Spectrometer (ICP-OES PerkinElmer Optima 2100DV). A Techcomp CT14D centrifuge (Shanghai, China) was used to separate the gels and solutions. XPS spectroscopy was recorded on a PHI-5000 spectrometer (Ulvac-Phi, Japan) in the range of 0–4000 eV.

## Supplementary information

Supplementary information.

## Data Availability

All data included in this study are available upon request by contact with the corresponding author.
