# Supplementary material for: High and fast adsorption of Cd(II) and Pb(II) ions from aqueous solutions by a waste biomass based hydrogel
Source: Sci Rep. 2020 Feb 24;10:3285. doi: 10.1038/s41598-020-60160-w (PMC7040188; doi:10.1038/s41598-020-60160-w)
Supplement: Supplementary file 1 — Supplementary information. [file 41598_2020_60160_MOESM1_ESM.pdf]

High and fast adsorption of Cd(II) and Pb(II) ions from aqueous solutions by a waste biomass based hydrogel

Mingyue Zhang*, Quanyu Yin, Xiaoming Ji, Fangling Wang, Xia Gao and Mingqin Zhao*

College of Tobacco Science, Flavors and Fragrance Engineering & Technology Research Center of Henan Province, Henan Agricultural University, Zhengzhou, 450002, P. R. China.

* Mingyue Zhang: mingyuezhang@henau.edu.cn

*Mingqin Zhao: zhaomingqin@126.com

Results

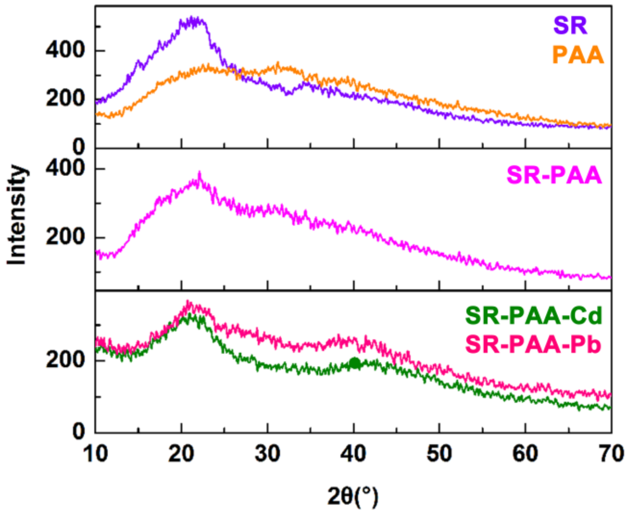

Fgure S1. XRD pattern of SR, PAA, SR-PAA, SR-PAA-Cd and SR-PAA-Pb.

Table S1. Summarization and assignments of the binding energies (BE) and atomic fractions (AF) of C 1s, O 1s and N 1s spectra of SR–PAA, SR–PAA–Cd and SR–PAA–Pb hydrogels.

Element	SR–PAA		SR–PAA–Cd		SR–PAA–Pb		Assignments
	BE/eV	AF/%	BE/eV	AF/%	BE/eV	AF/%	
C 1s	284.50	27.78	284.46	32.33	284.50	29.03	C–C, C–H
C 1s	285.00	15.23	285.00	14.88	285.00	16.83	C–N and C–O–C
C 1s	286.20	12.44	285.93	10.83	285.93	11.69	C–O, O–C–O and C=O
C 1s	288.05	9.54	288.46	8.91	288.55	9.34	$–COO^-$
Total C1s	–	64.99	–	66.95	–	66.89	–
O 1s	531.20	8.15	531.59	7.35	531.50	6.86	C–O and C–O–C
O 1s	532.20	6.60	532.14	6.81	532.20	5.66	C=O and O–C–O
O 1s	533.00	5.09	533.23	5.32	533.10	6.52	$–COO^-$
Total O1s	–	19.84	–	19.49	–	19.04	–
N 1s	399.62	10.11	399.76	7.17	400.05	7.60	N–H
N 1s	400.40	5.06	400.40	3.58	400.40	3.80	N–C
Total N1s	–	15.17	–	10.75	–	11.40	–

Table S2. Cd(II) and Pb(II) adsorption on SR, PAA and SR–PAA under the condition of 25 ℃, pH = 6, C_0=2.5 mmol L^{-1}.

Ions	SR/mmol L^{-1}	PAA/mmol L^{-1}	SR–PAA/mmol L^{-1}
Cd(II)	0.03	0.45	1.43
Pb(II)	0.33	1.69	2.03

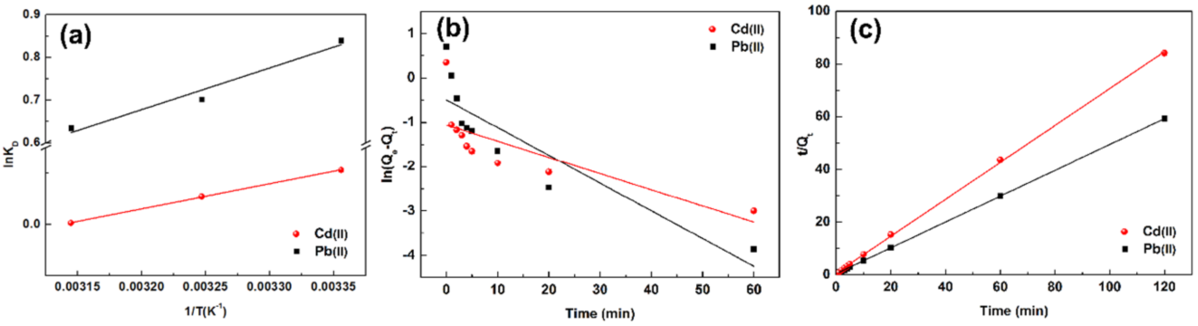

Figure S2. (a) Linear plots of $\ln K_D$ versus 1/T of Cd(II) and Pb(II) adsorption, (b) The pseudo-first-order and (c) The pseudo-second-order plots of metal ions adsorption data.

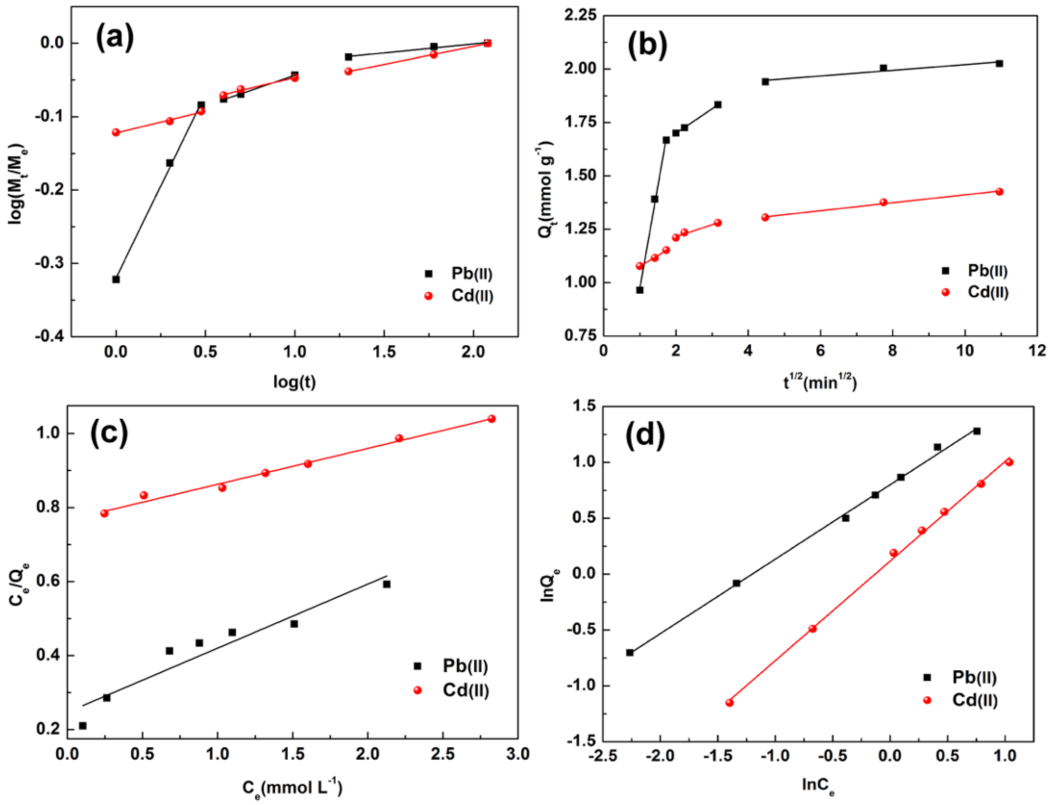

Figure S3. (a) Diffusion Kinetic, (b) The intra-particle diffusion kinetic, (c) Langmuir and (d) Freundlich plots of Cd(II) and Pb(II) ions adsorption.

Materials and methods

Preparation of PAA hydrogel

PAA was prepared according to the synthesis methods of SR–PAA, except no SR.

Experiments of effect of adsorption conditions

Effect of pH value on metal adsorption on SR-PAA was determined by soaking SR-PAA in solutions (2.5 mmol L^{-1}) at pH value of 2, 3, 4, 5 and 6 at 25 ℃, respectively. The optimum pH was confirmed as 6 and used throughout all other adsorption experiment. Experiments of temperature effect were carried out at 25, 35 and 45 ℃, respectively (pH = 6, C_0 = 2.5 mmol L^{-1}). Experiments of initial concentration effect were implemented with C_0 of 0.50, 1.00, 2.00, 3.00, 4.00 and 5.00 mmol L^{-1}, respectively (25 ℃, pH = 6). Effect of time on metal adsorption experiment was evaluated with soaking time of 1, 2, 3, 4, 5, 10, 20, 60 and 120 min, respectively (25 ℃, pH = 6, C_0 = 2.5 mmol L^{-1}). And the hydrogels that adsorption for 120 min were recorded as SR–PAA–Cd and SR–PAA–Pb, respectively. Soak time of all the experiments was 120 min except that effect of time experiments.

X-ray Diffraction Analysis

X-Ray diffraction (XRD) data were recorded on a Siemens D5005XRD diffractometer of ranges from 10 °to 70 °.
